# Speaker Sex Influences Processing of Grammatical Gender

**DOI:** 10.1371/journal.pone.0079701

**Published:** 2013-11-13

**Authors:** Michael S. Vitevitch, Joan Sereno, Allard Jongman, Rutherford Goldstein

**Affiliations:** 1 Department of Psychology, University of Kansas, Lawrence, Kansas, United States of America; 2 Department of Linguistics, University of Kansas, Lawrence, Kansas, United States of America; University of Akron, United States of America

## Abstract

Spoken words carry linguistic and indexical information to listeners. Abstractionist models of spoken word recognition suggest that indexical information is stripped away in a process called normalization to allow processing of the linguistic message to proceed. In contrast, exemplar models of the lexicon suggest that indexical information is retained in memory, and influences the process of spoken word recognition. In the present study native Spanish listeners heard Spanish words that varied in grammatical gender (masculine, ending in -o, or feminine, ending in -a) produced by either a male or a female speaker. When asked to indicate the grammatical gender of the words, listeners were faster and more accurate when the sex of the speaker “matched” the grammatical gender than when the sex of the speaker and the grammatical gender “mismatched.” No such interference was observed when listeners heard the same stimuli, but identified whether the speaker was male or female. This finding suggests that indexical information, in this case the sex of the speaker, influences not just processes associated with word recognition, but also higher-level processes associated with grammatical processing. This result also raises questions regarding the widespread assumption about the cognitive independence and automatic nature of grammatical processes.

## Introduction

The speech signal contains *linguistic information*, conveying phonological, semantic, and syntactic information about a word, and *indexical information*, conveying paralinguistic information about the speaker, including regional and economic background, emotional state, as well as age and gender [1]. Many models of spoken word recognition assume that lexical representations are stored in an abstract form in memory after a process of normalization has stripped away numerous sources of variability in the speech signal, including indexical information [2]-[3]. Alternative to these abstractionist models of spoken word recognition are models that suggest the lexicon contains numerous exemplar representations, each containing detailed information about the word and the speaker [4]-[6]. A convincing source of evidence for exemplar models of the lexicon is the many studies which demonstrate that changing the identity of the speaker during various phases of the experiment leads to reduced levels of performance in a variety of word recognition tasks compared to single-talker conditions [7]-[8].

If the lexicon does indeed use exemplar representations—containing both linguistic and indexical information—to process spoken words, then we should be able to observe influences of other aspects of indexical information at other levels of linguistic processing than have been previously observed. Previous studies typically changed the identity of the speaker, and measured the effect of this change using conventional word recognition tasks to assess the process of lexical access [*cf.*, 9].

In the present study, we examined if the sex of the speaker would influence a language process that occurs after lexical access, namely identifying the grammatical gender of a word. While the English language makes a grammatical distinction in number—singular versus plural (e.g., car vs. cars)—it does not make a distinction in grammatical gender. In languages that make gender distinctions, approximately a third of the world's languages, including Spanish, German, and Russian among many others [10], words are identified as being grammatically masculine, feminine, or in some cases neuter. Markers of grammatical gender in the words in a sentence must be appropriately applied to a word and must agree in order for a sentence to be grammatically correct in that language.

In the present study, we asked native speakers of Spanish to indicate whether the word they heard was grammatically masculine (e.g., piso; *floor*) or feminine (e.g., copa; *cup*). Crucially, male and female speakers produced both grammatically masculine and feminine words. If indexical information affects processes other than lexical access, then a “mismatch” between grammatical gender and the sex of the speaker should cause interference during the grammatical decision task, resulting in slower and less accurate responses in these conditions compared to conditions in which the grammatical gender of the word and the sex of the speaker “match.” Alternatively, if indexical information does not influence processing once lexical access has occurred, then the acoustic characteristics that distinguish a male from a female speaker should have no influence on the speed and accuracy with which listeners decide whether a word is grammatically masculine or feminine.

## Methods

### Participants

20 native Spanish speakers (6 male and 14 female) enrolled at the University of Kansas were paid $10 for their participation. None of the participants reported a history of speech or hearing disorders. All participants gave their written consent to participate in the experiment, which had been approved by the IRB of the University of Kansas.

### Materials

Forty words were unambiguously grammatically masculine (ending in /o/), and forty words were unambiguously grammatically feminine (ending in /a/). Each word contained 2 syllables and four phonemes (see Appendix S1). There was no difference in the frequency of occurrence for the masculine (*mean* = 110.78 occurrences per million, *sd* = 115.58) and feminine words (*mean* = 105.68 occurrences per million, *sd* = 110.47; *t* (78) = .20, *p* = .84), based on the frequency of occurrence counts in [11]. In addition, comparable numbers of stops, fricatives, and approximants appeared in the initial position of the masculine and feminine words.

A male and a female speaker—both native speakers of Spanish—produced all of the stimuli by speaking at a normal speaking rate and loudness in an IAC anechoic chamber into an ElectroVoice N/D767a microphone. Stimulus words were recorded digitally at a sampling rate of 22.05 kHz using a Marantz PMD 671 solid-state recorder. Each stimulus word was edited into an individual sound file, and all sound files were equated for amplitude without distorting the sound or changing the pitch of the words.

### Procedure

Participants were tested individually. Each participant was seated in front of an iMac computer running PsyScope 1.2.2 [12], which controlled the presentation of stimuli and the collection of responses. In each trial, a string of asterisks appeared on the computer screen for 500 ms to indicate the start of a trial. Participants then heard one of the randomly selected stimulus words through a set of Beyerdynamic DT 100 headphones at a comfortable listening level.

In the *speaker task*, listeners pressed a response button to indicate whether the word they heard was spoken by a man or a woman. Stick-figures resembling those used to indicate public toilets were used to label the response buttons (with male on the left for half of the participants and male on the right for half of the participants).

In the *grammatical gender task*, listeners pressed a response button to indicate whether the word they heard was grammatically masculine or grammatically feminine. The articles *el* (masculine) and *la* (feminine) were used to label the response buttons (with masculine on the left for half of the participants and masculine on the right for half of the participants). The two tasks were administered in counter-balanced order.

In each task, listeners received 160 trials, with each word being heard twice, once in a “matched” condition (grammatically masculine word produced by the male speaker, or grammatically feminine word produced by the female speaker), and once in a “mismatched” condition (grammatically masculine word produced by the female speaker, or grammatically feminine word produced by the male speaker). Counterbalanced lists were used, such that across participants each word appeared in each “matched” condition and each “mismatched” condition.

## Results

A repeated measures ANOVA was used to examine accuracy rates and response speed to “matched” conditions (the mean of grammatically masculine words produced by the male speaker, and grammatically feminine words produced by the female speaker) and to “mismatched” conditions (the mean of grammatically masculine words produced by the female speaker, and grammatically feminine words produced by the male speaker) in each task.

In the *speaker task* (i.e., indicate whether the voice saying the word was a male or female speaker), listeners responded quickly and accurately overall, but there was no significant difference in response speed between the “matched” (*mean* = 694.52 ms, *sd* = 102.15) and “mismatched” conditions (*mean* = 698.26 ms, *sd* = 107.44; *F* (1, 19) = .36, *p* = .56). The difference in accuracy between the “matched” (*mean* = 94.38%, *sd* = 4.21) and “mismatched” (*mean* = 94.31%, *sd* = 4.28) conditions was not significant either, *F* (1, 19) = .01, *p* = .92. Table 1 shows the mean response times and accuracy rates (and standard deviations for each measure) for each condition in the speaker task.

**Table 1:** Mean Response Times and Accuracy Rates for the Speaker Task.

Condition	Grammatical Gender	Gender of the Speaker	Response Time	Accuracy Rate
*Match*	*Masculine*	*Male*	*705ms (104)*	*94% (6)*
	*Feminine*	*Female*	*684ms (102)*	*95% (4)*
Mismatch	Masculine	Female	694ms (114)	95% (6)
	Feminine	Male	702ms (106)	94% (4)

Note: Italics indicate the values that contributed to the “matched” condition, and the non-italicized information indicates the values that contributed to the “mismatched” condition. Standard deviations are shown in parentheses.

However, when the same listeners were asked in the *grammatical gender task* to indicate whether the same words were grammatically masculine or feminine, a significant difference was observed between the “matched” and “mismatched” conditions for both response speed and accuracy. Table 2 shows the mean response times and accuracy rates (and standard deviations for each measure) for each condition in the grammatical gender task. Listeners were slower making decisions about the grammatical gender of the words when the grammatical gender and sex of the speaker “mismatched” (*mean* = 1027.64 ms, *sd* = 111.15) compared to when the grammatical gender and sex of the speaker “matched” (*mean* = 1001.71 ms, *sd* = 115.43; *F* (1, 19) = 17.42, *p*<.001, *Cohen*'*s d* = .23). Listeners were also less accurate making these decisions when the grammatical gender and sex of the speaker “mismatched” (*mean* = 85.88%, *sd* = 10.96) compared to when the grammatical gender and sex of the speaker “matched” (*mean* = 88.13%, *sd* = 9.58; *F* (1, 19) = 5.52, *p*<.05, *Cohen's d* = .22).

**Table 2:** Mean Response Times and Accuracy Rates for the Grammatical Gender Task.

Condition	Grammatical Gender	Gender of the Speaker	Response Time	Accuracy Rate
*Match*	*Masculine*	*Male*	*1005ms (111)*	*88% (10)*
	*Feminine*	*Female*	*997ms (124)*	*89% (9)*
Mismatch	Masculine	Female	1021ms (111)	84% (12)
	Feminine	Male	1033ms (115)	88% (10)

Note: Italics indicate the values that contributed to the “matched” condition, and the non-italicized information indicates the values that contributed to the “mismatched” condition. Standard deviations are shown in parentheses.

Statistical conventions suggest that *Cohen's d* [13] around .2 to .3 is considered a small effect, around .5 is considered a medium effect, and greater than .8 is considered a large effect. By these conventions, the effects observed in the present experiment are considered small in magnitude. However, the effect observed in the present experiment is comparable to the size of the effect of phonological markings of gender on response times in a grammatical gender task reported in [14]. Based on the means and standard deviations of the responses to the phonologically marked (*mean* = 657ms, *sd* = 104) and unmarked (*mean* = 679ms, *sd* = 106) stimuli reported in [14], we computed a *Cohen's d* of .21. Given the small effect size observed in [14] for phonological information in a grammatical gender task, it should not be surprising that the effect of acoustic characteristics associated with male and female speakers in the grammatical gender task in the present experiment—though statistically significant—is also small in magnitude.

## Discussion

The results of the present study show that native-Spanish listeners were slower and less accurate making grammatical gender decisions about unambiguously grammatically masculine words (ending in /o/), and unambiguously grammatically feminine words (ending in /a/) when the sex of the speaker “mismatched” the grammatical gender of the words (i.e., a female speaker producing a grammatically masculine word) than when the speaker “matched” the grammatical gender of the words (i.e., a female speaker producing a grammatically feminine word). This difference was not due to any differences between speakers since native Spanish listeners, hearing the same stimuli, showed no preference when indicating the gender of the speaker. Together, these findings are consistent with previous research showing that changes in indexical information can influence the processing of linguistic information [15]-[17]. Such findings have been often used to argue in favor of exemplar models of the mental lexicon (see [18] for an overview), as an alternative to abstractionist models of lexical processing.

Although the present results, like previous results, can be accounted for by exemplar models of the mental lexicon, the present findings differ from previous studies examining how indexical information influences the processing of linguistic information in several important ways. First, previous studies have typically demonstrated that *changes* in indexical information affect lexical processing. However, in the present study, we demonstrated that more *fine-grained* information contained in the indexical portion of the speech signal—the identity of the speaker as male or female, as opposed to just a change in the speaker—influences the processing of linguistic information in more complex ways.

In both tasks used in the present study, listeners heard a male and female speaker. If processing was affected simply by the fact that two different speakers were employed in the task, then processing deficits should have been observed in both tasks. This was not the case; responses in the speaker task were made very quickly and accurately across conditions. Only in the grammatical decision task did we observe a rather specific deficit in performance: performance was slower and less accurate when the sex of the speaker “mismatched” the grammatical gender of the word being spoken. Thus, the present study demonstrates that it is not simply *changes* in indexical information that disrupt processing of linguistic information, but that the *actual information* conveyed in the indexical portion of the speech signal can affect processing of linguistic information in very specific ways. The distinction we describe may appear subtle, but this distinction is one that is theoretically substantial, making this study a unique and important contribution to the literature.

The present results are also of theoretical importance because the influence of “lower level” acoustic information on “higher level” linguistic information (i.e., the concept of grammatical gender) is in the opposite direction of what one might expect from the perspective of embodied cognition, also known as *grounded cognition* [19]. Numerous behavioral studies have shown that the activation of higher-level conceptual information can influence performance of lower-level perceptions or actions (see [19] for a review of such studies). In contrast, the present results show that acoustic information in the speech signal that corresponds to the identity of the speaker as male or female influenced the processing of higher-level information that corresponds to the linguistic construct of grammatical gender.

This is not to say, however, that grounded cognition is not involved in the processing of acoustic information or grammatical gender. From the perspective of grounded cognition one might predict that the higher-level concept of the gender of the *listener* might influence their performance in either the speaker task or the grammatical gender task, such that performance in these tasks would be (further) facilitated if the gender of the listener matched the gender of the speaker and the grammatical gender of the words being spoken. Unfortunately, the sample of listeners in the present experiment was too small and not balanced in the number of males (*n* = 6) and females (*n* = 14) to allow us to assess this hypothesis. A future study using a larger and more balanced sample (in terms of gender of the listeners), and perhaps a more sensitive measure (such as eye-tracking measures) might be able to better assess the influence of grounded cognition on processing.

Another way in which the present findings differ from previous studies that examined how indexical information influences the processing of linguistic information is that the present results demonstrate that indexical information can influence grammatical processes. Previous studies have typically examined the influence of indexical information such as number of talkers or speaking rate variability on the memory of verbal materials and the performance on spoken word recognition tasks that assess the process of lexical access, whereas we observed influences of indexical information on a later stage of processing dealing with grammatical gender. This suggests that the influence of indexical information on processing may be more wide-spread than previous findings indicate.

Finally, and more broadly, the result of this experiment addresses several widespread assumptions about the cognitive independence and automatic nature of grammatical processes [20]-[22]. The present experiment shows that acoustic information associated with the sex of a speaker interacts with the processing of the linguistic information associated with grammatical gender, suggesting that grammatical processing may not be as cognitively distinct from outside sources of information as previously held.

## Supporting Information

Appendix S1 The Spanish words and their English translations used in the experiments.
(DOCX)
