# Supplementary material for: Speaker Sex Influences Processing of Grammatical Gender
Source: PLoS One. 2013 Nov 13;8(11):e79701. doi: 10.1371/journal.pone.0079701 (PMC3827416; doi:10.1371/journal.pone.0079701)
Supplement: Appendix S1 — The Spanish words and their English translations used in the experiments. (DOCX) [file pone.0079701.s001.docx]

**Appendix**

| **Feminine Words** | | **Masculine Words** | |
| --- | --- | --- | --- |
| **Spanish** | **English translation** | **Spanish** | **English translation** |
| beca  boda  copa  daga  duna  faja  fama  fila  foca  fosa  fuga  gira  gota  lana  lata  liga  lima  lona  losa  luna  lupa  mina  mora  nata  nuca  pata  pena  pesa  pila  rana  rima  risa  roca  ruta  sala  seda  soga  suma  tapa  tela | grant  wedding  cup  dagger  dune  band  fame  line  seal  grave  escape  tour  drop  wool  can  league  lime  canvass  flagstone  moon  magnifying glass  mine  blackberry  cream  nape  leg  shame  weight  battery  frog  rhyme  laugh  rock  route  livingroom  silk  rope  sum  lid  fabric | baño  beso  dato  dedo  fajo  faro  filo  foco  foro  foso  gato  gozo  kilo  lago  lazo  lino  lobo  lodo  loro  lujo  luto  mito  moho  muro  nido  pato  pelo  pico  piso  rabo  ramo  rayo  reto  robo  saco  sapo  seno  seto  tiro  tubo | bath  kiss  fact  finger  bundle  lighthouse  edge  focus  court  ditch  cat  joy  kilo  lake  bow  linen  wolf  mud  parrot  luxury  mourning  myth  mold  wall  nest  duck  hair  beak  floor  tail  bunch  ray  challenge  theft  sack  toad  chest  fence  shot  pipe |
